# Comparative risk of malignancies and infections in patients with rheumatoid arthritis initiating abatacept versus other biologics: a multi-database real-world study

**DOI:** 10.1186/s13075-019-1992-x

**Published:** 2019-11-08

**Authors:** Teresa A. Simon, Maarten Boers, Marc Hochberg, Nicole Baker, Mary L. Skovron, Nitesh Ray, Sanket Singhal, Samy Suissa, Andres Gomez-Caminero

**Affiliations:** 1 Bristol-Myers Squibb, Princeton, NJ USA; 2 Amsterdam University Medical Centers, Vrije Universiteit, Amsterdam, The Netherlands; 3 University of Maryland School of Medicine, Baltimore, MD USA; 4 Mu Sigma, Bengaluru, Karnataka India; 5 McGill University, Montreal, Quebec Canada

**Keywords:** Abatacept, Real-world data, Claims, Infections, Malignancies, Rheumatoid arthritis

## Abstract

**Background:**

Patients with rheumatoid arthritis (RA) are at an increased risk of developing certain cancers and infections compared with the general population. Biologic and targeted synthetic disease-modifying antirheumatic drugs (b/tsDMARDs) are effective treatment options for RA, but limited evidence is available on the comparative risks among b/tsDMARDs. We assessed the risk of malignancies and infections in patients with RA who initiated abatacept versus other b/tsDMARDs in a real-world setting.

**Methods:**

This retrospective, observational study used administrative data from three large US healthcare databases (MarketScan, PharMetrics, and Optum) to identify patients treated with abatacept or other b/tsDMARDs. In both groups, age-stratified incidence rates (IRs) with 95% confidence intervals (CIs) were calculated for total malignancy and hospitalized infections; propensity score matching and Cox proportional hazards regression models were used to estimate hazard ratios (HRs) with 95% CIs for total malignancy, lung cancer, lymphoma, breast cancer, non-melanoma skin cancer (NMSC), hospitalized infections, opportunistic infections, and tuberculosis (TB), both within individual databases and in meta-analyses across the three databases.

**Results:**

A rounded total of 19.2, 13.6, and 4.2 thousand patients initiating abatacept and 55.3, 40.8, and 13.8 thousand initiating other b/tsDMARDs were identified in the MarketScan, PharMetrics, and Optum databases, respectively. The IRs for total malignancy and hospitalized infections were similar between the two groups in each age stratum. In meta-analyses, total malignancy risk (HR [95% CI] 1.09 [1.02–1.16]) of abatacept versus other b/tsDMARDs was slightly but statistically significantly increased; small, but not statistically significant, increases were seen for lung cancer (1.10 [0.62–1.96]), lymphoma (1.27 [0.94–1.72]), breast cancer (1.15 [0.92–1.45]), and NMSC (1.10 [0.93–1.30]). No significant increase in hospitalized infections (0.96 [0.84–1.09]) or opportunistic infections (1.06 [0.96–1.17]) was seen. For TB, low event counts precluded meta-analysis.

**Conclusions:**

In this real-world multi-database study, the risks for specific cancers and infections did not differ significantly between patients in the abatacept and other b/tsDMARDs groups. The slight increase in total malignancy risk associated with abatacept needs further investigation. These results are consistent with the established safety profile of abatacept.

## Introduction

Patients with rheumatoid arthritis (RA) not only bear a high burden of disease [1–4], but are also at an increased risk of developing infections and certain cancers, particularly lymphomas and lung cancer, compared with the general population [5–9]. Given the chronic nature of RA and the requirement for prolonged treatment, it is important for physicians to consider the long-term safety of different treatments in addition to their efficacy when making treatment decisions.

Biologic and targeted synthetic disease-modifying antirheumatic drugs (b/tsDMARDs) are effective treatments for RA. However, current evidence is inconsistent as to whether the use of b/tsDMARDs generally or specific b/tsDMARDs increases the risk of malignancies and infections in the RA population. Higher rates of malignancies [10] and infections [10, 11] have been observed with tumor necrosis factor (TNF) inhibitors when compared with placebo or conventional synthetic (cs) DMARDs in some studies. Other studies either showed no increased risk with bDMARDs versus csDMARDs [12–15] or were inconclusive [16]. When safety risks across different bDMARDs were examined, similar rates of malignancies and infections with abatacept and adalimumab were reported in a phase III randomized clinical trial [17]. Furthermore, a systematic literature review informing the 2016 update of the European League Against Rheumatism recommendations recognized a lack of evidence on the risks of malignancies, tuberculosis (TB), and herpes zoster virus across bDMARDs [18].

Abatacept, a selective T cell co-stimulation modulator approved for the treatment of RA, has a mechanism of action that is fundamentally different from that of other b/tsDMARDs [19–22]. Several clinical trials have demonstrated the efficacy, tolerability, and safety profile of abatacept [23–26]. Low incidence rates (IRs) for malignancies and infections have been reported with abatacept treatment in clinical trials [14, 23, 26, 27]; however, the risks associated with the long-term use of abatacept versus other b/tsDMARDs in the real-world setting need further investigation [28]. This study used information from three large US healthcare claims databases to evaluate the risk of malignancies and infections in patients with RA treated with either abatacept or other b/tsDMARDs in a real-world setting.

## Methods

### Study design and data sources

Data from three US administrative healthcare databases were included in this retrospective, real-world, observational study: Truven MarketScan Commercial and Supplemental Medicare (MarketScan), IMS PharMetrics (PharMetrics), and Optum Clinformatics Data Mart (Optum). As of September 2014, the MarketScan database had information on over 70 million privately insured patients aged under 65 years and approximately 6 million patients aged 65 years or older receiving Medicare coverage from 2006 onwards. The PharMetrics database contains claims information on approximately 55 million patients from over 90 managed care plans and other sources starting from 2007. The Optum database contains claims information and enrollment data on approximately 35 million patients from 2002 onwards. Available data in all three databases included demographic variables, diagnostic codes, and treatment information.

### Patient population

Adult patients (aged ≥ 18 years) with RA who initiated abatacept or other b/tsDMARD treatments (index date) from July 1, 2006, to September 30, 2014, and had 180 or more days of continuous health plan enrollment before the index date were eligible for inclusion. Identification of patients with RA was based on MacLean’s criteria [29], which requires two or more inpatient or outpatient International Classification of Diseases, Ninth Revision, Clinical Modification (ICD-9-CM) diagnosis codes for RA (714.xx) in the patient’s history (prior to and including the index date) or within 6 months of the index date. Patients were assumed to have initiated a treatment when there was no claim for that particular drug in the 180-day period before the index date. Thus, patients in this study could be first-time initiators for that drug, could have stopped that treatment > 180 days before the index date and subsequently restarted, or could be switching from another b/tsDMARD. Patients who initiated abatacept at any point during the study period were included in the abatacept group; those who initiated more than one b/tsDMARD (excluding abatacept) were assigned to the first drug initiated. Treatments were identified using national drug codes for dispensed medications and procedure codes for injection or infusion. Other b/tsDMARDs were infliximab, etanercept, adalimumab, certolizumab pegol, golimumab, tocilizumab, rituximab, tofacitinib, and anakinra.

### Study outcomes

All patients identified for the abatacept and other b/tsDMARDs groups were included in the analyses within each database and meta-analyses across all three databases. ICD-9-CM diagnosis codes were used to identify the safety outcomes investigated: total malignancy (including non-melanoma skin cancer [NMSC]), lung cancer, lymphoma, breast cancer, NMSC, hospitalized infections, opportunistic infections, and TB. For each outcome computation, patients who had at least one ICD-9-CM code for that outcome in the 6-month period before the index date were excluded from the analysis of that particular outcome.

All malignancy outcomes were identified by ICD-9-CM codes. The follow-up period extended from 180 days after the index date until the occurrence of a malignancy, end of enrollment in the database, or end of data collection, whichever occurred first. A latency period of 180 days was included to allow for plausible induction time for the appearance of a malignancy associated with a new treatment and to avoid protopathic bias. The latency period and any malignancies that occurred during the latency period were not included in the analysis of malignancy outcomes.

For infection-related outcomes, the follow-up period extended from the index date to the occurrence of the infection of interest, end of the initiated treatment plus 90 days, end of enrollment in the database, or end of data collection, whichever occurred first. Infection-related outcomes were identified using ICD-9-CM diagnosis codes in conjunction with an inpatient stay or an outpatient physician visit procedure code. Opportunistic infections were identified using ICD-9-CM codes for primary TB infection, TB, herpes zoster virus, dermatophytosis, dermatomycosis, candidiasis, coccidioidomycosis, histoplasmosis, blastomycosis, and opportunistic or other mycoses. Classification of a case of TB required (1) prescription of a medication regimen including pyrazinamide, (2) an ICD-9-CM code for TB and prescriptions for two anti-TB medications excluding pyrazinamide, or (3) an ICD-9-CM code for TB and an order for a TB diagnostic test [30].

### Statistical analyses

Descriptive statistics were used to analyze baseline demographic information, co-morbid conditions, and concomitant medication use. For each outcome of interest, the IR and hazard ratio (HR), with 95% confidence intervals (CIs), were calculated. IRs were calculated within each individual database by dividing the number of events for each outcome by the total person-time at risk, stratified by age group (18–64, 65–74, and ≥ 75 years). A Poisson distribution was assumed to compute 95% CIs for IRs.

To compare the safety events of abatacept with those of other b/tsDMARDs, HRs with 95% CIs were calculated using multivariate Cox proportional hazards regression models. The calculations were performed separately for each individual database and across all three databases as a meta-analysis according to published methods [31–33]. Two methods were used to control for confounding factors: a propensity score-matched analysis and a propensity score-adjusted analysis. A propensity score was developed for each patient by index date within each database. Propensity scores were estimated separately within each database by logistic regression analyses incorporating measured potential predictors of therapy (Additional file 1: Tables S1–S6) as independent variables in the regression model and comparison group status as the outcome. In the matched analyses, each patient in the abatacept group was matched to up to two patients from the comparison group to achieve a balance between comparison groups in terms of all identified predictors of abatacept initiation. Propensity score matching was used for the analysis of all outcomes except TB. For the analysis of the outcome of TB, a published algorithm developed by Calderwood et al. [30] was applied to the propensity score-adjusted model in which the variable imbalances between the groups were examined using Cohen’s *d* test and adjusted for in the final outcome model. The propensity score-adjusted model was used instead of the propensity score matching model to maximize the number of patients in the TB analysis. Additional details regarding the propensity score-matched and propensity score-adjusted analyses can be found in Additional file 1 (statistical analyses: variable selection for models).

To validate the level of specificity for the outcome identification, a sensitivity analysis was performed in which two ICD-9-CM codes for opportunistic infections were required in order to be counted as an event. The date of the first ICD-9-CM code claim was used as the event date for computation.

## Results

### Patient disposition

**Fig. 1:**
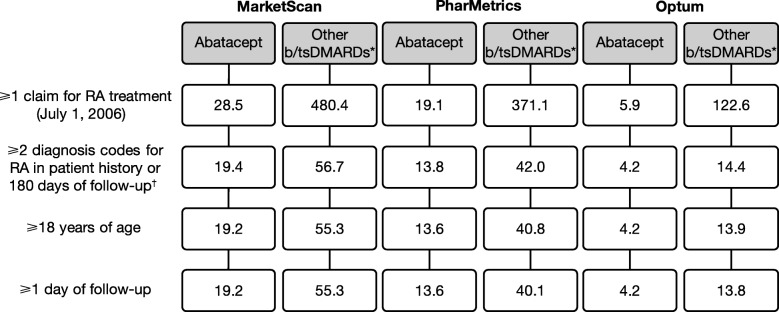
Patient disposition. All numbers expressed are in thousands. *Excludes abatacept. ^†^Based on MacLean’s positive predictive value of an administrative data-based algorithm for the identification of patients with RA [29]. b/tsDMARDs, biologic or targeted synthetic disease-modifying antirheumatic drugs; RA, rheumatoid arthritis

A rounded total of 19.2, 13.6, and 4.2 thousand patients initiating abatacept and 55.3, 40.8, and 13.8 thousand initiating other b/tsDMARDs were identified in the MarketScan, PharMetrics, and Optum databases, respectively (Fig. 1; Additional file 1: Table S7). After matching, 17.5, 12.1, and 3.4 thousand patients initiating abatacept and 32.3, 21.1, and 5.6 thousand initiating other b/tsDMARDs were included from the MarketScan, PharMetrics, and Optum databases, respectively (Table 1).

**Table 1:** Demographics and baseline characteristics of patients in the matched abatacept and other b/tsDMARDs groups

	MarketScan		PharMetrics		Optum	
	Abatacept (*n* = 17,517)	Other b/tsDMARDs* (*n* = 32,277)	Abatacept (*n* = 12,120)	Other b/tsDMARDs* (*n* = 21,145)	Abatacept (*n* = 3354)	Other b/tsDMARDs* (*n* = 5604)
Female, %	82	83	80	81	82	82
Age in years at index date, mean (SD)	55 (13)	54 (13)	53 (12)	52 (12)	51 (11)	51 (11)
Co-morbid conditions during the baseline period, %						
Malignancy	4.8	4.2	4.5	3.7	3.0	1.9
Cardiovascular disease^†^	22	19	21	17	21	17
Hospitalized infections	3.2	2.3	3.5	2.6	3.7	2.9
Other autoimmune diseases^‡^	17	15	20	18	17	16
Co-medications^§^, %						
csDMARDs	56	53	62	58	66	61
b/tsDMARDs	48	15	53	17	59	21
Glucocorticoids	54	53	61	59	71	71

*b/tsDMARDs* biologic or targeted synthetic disease-modifying antirheumatic drugs, *csDMARDs* conventional synthetic disease-modifying antirheumatic drugs, *SD* standard deviation

*Excludes abatacept

^†^Includes ischemic heart disease; diseases of pulmonary circulation; other forms of heart disease; cerebrovascular disease; diseases of the arteries, arterioles, and capillaries; diseases of the veins and lymphatics; and other diseases of the circulatory system

^‡^Includes psoriatic arthropathy, other psoriasis, diabetes mellitus, multiple sclerosis, systemic lupus erythematosus, vitiligo, toxic diffuse goiter without mention of thryrotoxic crisis or storm, chronic lymphocytic thyroiditis, corticoadrenal insufficiency, acquired hemolytic anemias, immune thrombocytopenic purpura, chronic glomerulonephritis, cirrhosis of liver without mention of alcohol, celiac disease, regional enteritis, ulcerative enterocolitis, postinflammatory pulmonary fibrosis, giant cell arteritis, sicca syndrome, systemic sclerosis, alopecia areata, and urticaria

^§^Includes medications taken within 180 days before the index date

### Baseline characteristics

Baseline characteristics were generally well balanced across both groups (Additional file 1: Table S7). However, in each database, patients in the abatacept group were slightly older, more likely to be female, less likely to have another autoimmune disease, and more likely to have had exposure to b/tsDMARDs and csDMARDs at baseline (within 180 days before the index date) compared with those in the other b/tsDMARDs group (Additional file 1: Table S7). Patients were also more likely to have had a previous claim for hospitalized infections or cardiovascular disease in the abatacept group than those in the other b/tsDMARDs group. Distribution of index treatments among patients in the b/tsDMARDs group was similar across the three databases, with TNF inhibitors being the most commonly used (Additional file 1: Table S8). In matched patients, all demographics and baseline characteristics were comparable except for exposure to b/tsDMARDs at baseline (within 180 days before the index date), which was greater in the abatacept group compared with the other b/tsDMARD group (Table 1).

### Outcomes

#### Malignancy outcomes

**Fig. 2:**
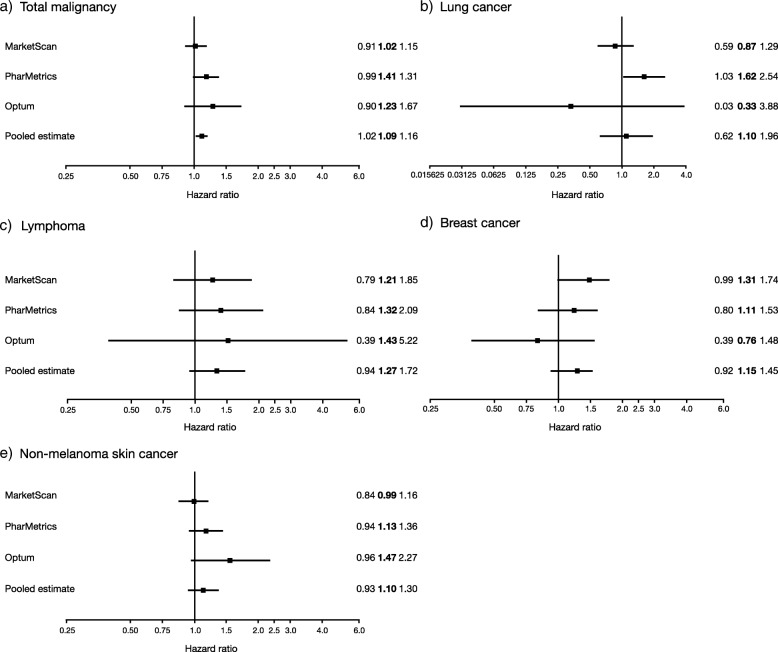
Hazard ratios* for malignancies^†^ in abatacept versus other b/tsDMARD Initiators: a) total malignancy, b) lung cancer, c) lymphoma, d) breast cancer, e) non-melanoma skin cancer. *Error bars represent 95% CIs. ^†^One ICD-9-CM code. b/tsDMARDs, biologic or targeted synthetic disease-modifying antirheumatic drugs; CI, confidence interval; HR, hazard ratio; ICD-9-CM, International Classification of Diseases, Ninth Revision, Clinical Modification

The IRs for total malignancy were slightly higher in the abatacept-treated patients relative to the other b/tsDMARDs group in each age stratum (Additional file 1: Table S9). The regression analyses in individual databases showed no significant difference in the risk of total malignancy in abatacept initiators compared with other b/tsDMARDs initiators (Fig. 2a), although IRs were consistently slightly higher with abatacept versus other b/tsDMARDs across all databases. However, in the meta-analysis, a small but significant increase in the risk of total malignancy (HR [95% CI] 1.09 [1.02–1.16]) associated with abatacept versus other b/tsDMARDs emerged. An evaluation of all malignancies was performed, and no one type of malignancy was found to be more prevalent in abatacept compared with other b/tsDMARDs initiators (Additional file 1: Table S10).

The regression analyses for a single diagnosis of prespecified malignancy types showed no significant difference in the abatacept versus other b/tsDMARDs initiators in any database, with one exception (Fig. 2). A small but significant increase in the risk of lung cancer with abatacept versus other b/tsDMARDs was identified in the PharMetrics database (HR [95% CI] 1.62 [1.03–2.54]). The meta-analyses showed small, but not statistically significant, increases in the risk of lung cancer (Fig. 2b; HR [95% CI] 1.10 [0.62–1.96]), lymphoma (Fig. 2c; 1.27 [0.94–1.72]), breast cancer (Fig. 2d; 1.15 [0.92–1.45]), and NMSC (Fig. 2e; 1.10 [0.93–1.30]) with abatacept versus other b/tsDMARDs, consistent with that seen for the overall malignancy rate.

#### Infection-related outcomes

**Fig. 3:**
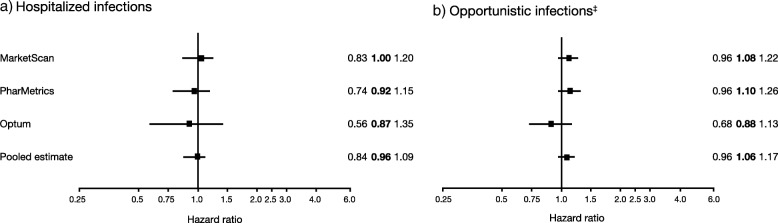
Hazard ratios* for a) hospitalized infections† and b) opportunistic infections in abatacept versus other b/tsDMARD initiators. Similar risk of TB was observed among abatacept and other b/tsDMARDs initiators in the MarketScan (HR [95% CI] 1.93 [0.45–8.32]) and PharMetrics (1.73 [0.17–17.63]) databases. It was not possible to analyze TB as an outcome in the Optum database because there were no events in the abatacept group and only two in the other b/tsDMARDs group. A meta-analysis of the outcome of TB was not performed due to low numbers. *Error bars represent 95% CIs. †Data represent hospitalizations associated with an infection as the primary diagnosis (one ICD-9-CM code). b/tsDMARDs, biologic or targeted synthetic disease-modifying antirheumatic drugs; CI, confidence interval; HR, hazard ratio; ICD-9-CM, International Classification of Diseases, Ninth Revision, Clinical Modification; IR, incidence rate; TB, tuberculosis

The IRs for hospitalized infections were slightly lower in the abatacept-treated patients compared with the b/tsDMARDs group overall (Additional file 1: Table S11) and by age strata (Additional file 1: Table S12). The analyses of individual databases showed no significant difference in the risk of hospitalized infections in abatacept initiators compared with other b/tsDMARDs initiators (Fig. 3a); this finding was confirmed by the results from the meta-analysis (HR [95% CI] 0.96 [0.84–1.09]).

For opportunistic infections, the regression analyses in individual databases and the meta-analysis (HR [95% CI] 1.06 [0.96–1.17]) showed no significant difference in risk in abatacept initiators relative to b/tsDMARDs initiators (Fig. 3b). The IRs for opportunistic infections were slightly higher in the abatacept-treated patients compared with the b/tsDMARDs group (Additional file 1: Table S11). In the sensitivity analysis requiring two ICD-9-CM codes, a small but significantly increased opportunistic infection risk with abatacept was observed in the PharMetrics database (HR [95% CI] 1.30 [1.07–1.58]). The results for the MarketScan and Optum databases (data not shown) were comparable with those of the primary analysis.

Applying the Calderwood algorithm [30] to the TB outcome analysis reduced the total number of events from 160, 104, and 40 to 10, 4, and 0 in the MarketScan, PharMetrics and Optum databases, respectively. Similar risk of TB was observed among abatacept and other b/tsDMARDs initiators in the MarketScan (HR [95% CI] 1.93 [0.45–8.32]) and PharMetrics (1.73 [0.17–17.63]) databases. It was not possible to analyze TB as an outcome in the Optum database because there were no events in the abatacept group and only two in the other b/tsDMARDs group. Thus, a meta-analysis of the outcome of TB was not performed. The IRs for tuberculosis varied across databases (Additional file 1: Table S11).

## Discussion

In this real-world study of safety outcomes in patients with RA using data from three large US healthcare databases, a slight increase in the risk of total malignancy associated with abatacept versus other b/tsDMARD treatment was observed. Meta-analyses of specific malignancy types (lung cancer, lymphoma, breast cancer, or NMSC) showed similar trends, but the differences were not statistically significant. The risks of hospitalized infections, opportunistic infections, and TB were similar in patients receiving abatacept as in those receiving other b/tsDMARDs.

Evidence is inconsistent as to whether the use of abatacept, compared with other b/tsDMARDs, is associated with an increased risk of malignancy [27, 28, 34–36]. Contrary to the results of previous population-based cohort studies, which included evaluations of all NMSC [35] and squamous cell skin cancer [37], no significantly increased risk of NMSC was observed with abatacept compared with other b/tsDMARDs in this study. The results of this study are consistent with those from a meta-analysis of clinical trials that showed b/tsDMARDs, including abatacept, were not significantly associated with an increased malignancy risk compared with csDMARDs or placebo [36]. Previous interventional trials and real-world analyses of abatacept demonstrated a similar malignancy risk of abatacept compared with placebo or other comparators [27, 28, 34]. There may be several reasons for the small but statistically significantly increased risk of overall malignancy seen with abatacept treatment, including the unique upstream mechanism of action of abatacept and differences in patient characteristics. Abatacept inhibits the CD80/CD86:CD28 costimulatory signal required for full T cell activation and as such may alter the immune responses to tumors, as well as dampening pathogenic autoimmunity [38]. The finding of increased total malignancy risk needs further investigation but should be considered with the following limitations in mind. There were some noticeable differences in baseline characteristics, such as age, sex, percentage of patients with other autoimmune diseases, and, particularly, prior exposure to b/tsDMARDs and csDMARDs between the abatacept and other b/tsDMARDs groups, which may have had an impact on the malignancy outcome. Propensity score matching was used in our study to control for confounding factors; however, residual confounding from unmeasured factors, such as clinical characteristics or smoking history, could not be ruled out. Prior exposure to b/tsDMARDs is particularly important for residual confounding considerations, as the treatments included for exposure analysis were the same as those included in the comparator group (other b/tsDMARDs) for the outcome analysis. A higher percentage of patients had prior exposure to b/tsDMARDs in the abatacept (49–58%) versus the comparator (14–19%) group. It could be speculated that patients who initiated abatacept may be at a more advanced disease stage and therefore received a greater number of prior therapies, compared with those who initiated other b/tsDMARDs, which was not adequately controlled for in our analysis. Rather than matching on this differential timing of cohort entry, we adjusted for these imbalances, which may not have fully removed the bias from the imbalance in prior exposure to other b/tsDMARDs [39]. This is particularly the case when attributing malignancies occurring after abatacept use to abatacept exposure as prior use of other b/tsDMARDs may also contribute to the occurrence of malignancies. In this study, a latency period of 180 days was used in an effort to address this challenge. Medications and co-morbid conditions that may influence malignancy risk were assessed during the 180-day baseline period, but changes were not assessed during the follow-up period. In addition, the short latency period of 180 days and follow-up period of 747–970 days may be insufficient for the study of cancer.

An increased risk of opportunistic infections in patients with RA receiving tumor necrosis factor inhibitors compared with those receiving csDMARDs was observed in a systematic review of 87 articles and 40 abstracts and a real-world observational study of the Corrona registry [40, 41]. However, limited comparative data are available for abatacept and other b/tsDMARDs [41]. This study suggests that risk of opportunistic infections is similar in patients receiving abatacept as in those receiving other b/tsDMARDs. In the sensitivity analysis, an elevated risk for opportunistic infection for abatacept was observed in one of the three databases. This observation may be due to the imbalances between the groups, such as greater co-medication differences in abatacept versus other b/tsDMARDs initiators in the PharMetrics database compared with those in other databases (Additional file 1: Table S7). The similar hospitalized infection risk in this study is consistent with the findings from previous studies comparing b/tsDMARDs including abatacept with csDMARDs [14, 42] and when comparing abatacept with other b/tsDMARDs [43].

Previous research has shown that ICD-9-CM codes alone may not yield a satisfactory positive predictive value for TB identification [44]. Thus, a published algorithm [30] was applied to increase the specificity of the TB identification in this study. The risk of TB was similar in patients receiving abatacept as in those receiving other b/tsDMARDs; however, this was not statistically significant. The comparable risk for TB with abatacept and other b/tsDMARDs observed in our study is consistent with the previously reported low IRs for TB in patients treated with b/tsDMARDs, including abatacept, using data from clinical trials and national registries, when correct screening and prophylaxis was applied [27, 45]. The low event count in the Optum database and lack of sufficient data for meta-analysis in our study preclude further conclusion, and further assessment of TB risk in larger studies is warranted.

Further limitations of this study should be considered. Since treatment was determined from the prescribing or dispensing records in each database, adherence to any of the RA treatments cannot be confirmed. As with any observational study based on claims data, identification of medical events or baseline co-morbid conditions was limited to data that were captured as part of the claim. Furthermore, the data captured in the databases were not primarily collected for research purposes; thus, diagnostic codes and algorithms were utilized to identify outcomes but cannot serve as a confirmation of the outcome. Finally, as both biologic-experienced and biologic-naïve patients were analyzed together, due to latency, it was not possible to eliminate the influence of prior therapy on malignancy risk. Despite these limitations, the sample size and, therefore, the ability to detect potential effects were maximized in this study by including data from three large claims databases.

## Conclusions

In this analysis of three large real-world US claims databases, abatacept initiators had a slightly increased risk of total malignancy compared with initiators of other b/tsDMARDs. Meta-analyses showed similar, but not statistically significant, trends for lung cancer, lymphoma, breast cancer, and NMSC. However, due to the imbalance in prior b/tsDMARD use between the groups, it was not possible to rule out residual confounding despite statistical adjustment or to identify the potential effects of prior b/tsDMARD therapy. The risks for hospitalized infections, opportunistic infections, and TB were similar in patients with RA who initiated abatacept and those who initiated other b/tsDMARDs. The findings of this analysis are consistent with the established safety profile of abatacept.

## Supplementary information

**Additional file 1:.** Statistical analyses: variable selection for models. Supplementary tables. (DOCX 39 kb)

## Data Availability

Bristol-Myers Squibb policy on data sharing may be found at https://www.bms.com/researchers-and-partners/clinical-trials-and-research/disclosure-commitment.html.

## Abbreviations

b/tsDMARD: Biologic and targeted synthetic disease-modifying antirheumatic drug
CI: Confidence interval
csDMARD: Conventional synthetic disease-modifying antirheumatic drug
HR: Hazard ratio
ICD-9-CM: International Classification of Diseases, Ninth Revision, Clinical Modification
IR: Incidence rate
NMSC: Non-melanoma skin cancer
py: Person-years
RA: Rheumatoid arthritis
SD: Standard deviation
TB: Tuberculosis
TNF: Tumor necrosis factor
