# Supplementary material for: Comparative risk of malignancies and infections in patients with rheumatoid arthritis initiating abatacept versus other biologics: a multi-database real-world study
Source: Arthritis Res Ther. 2019 Nov 8;21:228. doi: 10.1186/s13075-019-1992-x (PMC6839238; doi:10.1186/s13075-019-1992-x)
Supplement: Supplementary file 1 — Additional file 1:. Statistical analyses: variable selection for models. Supplementary tables. (DOCX 39 kb) [file 13075_2019_1992_MOESM1_ESM.docx]

**ADDITIONAL FILE 1**

***Statistical analyses: variable selection for models***

Propensity score–adjusted analysis

- Variables highlighted in red were not considered significant for propensity score generation (*P*<0.2 for model entry and model retention).
- The treatment variable and propensity score are included as predictors in the final model.

Propensity score–matched analysis

- Variables highlighted in red were not considered significant for propensity score generation (*P*<0.2 for model entry and model retention).
- Trimming was performed to remove patients with the lowest and highest 2.5% of propensity scores. Then, 1:2 greedy matching was performed with replacement and 8:4 digit matching on propensity score.
- Cohen’s D test was used to get the imbalanced covariates. Any variable that had a standardized difference of means >0.1 was identified as imbalanced. If an imbalanced variable was identified, the entire process was repeated until the model was balanced.
- The treatment variable and imbalanced variables are included as predictors in the final model.

**Table S1.** MarketScan: propensity score–adjusted analysis

| Independent predictors considered for propensity score generation | Sex, age at index date, year of index date, any bDMARD, csDMARDs, MTX, IV antibiotics, corticosteroids, NSAIDs, hypertension, diabetes, hospitalized infections, malignancy, lymphoma, COPD, asthma, chronic kidney disease, leukopenia, neutropenia, peripheral artery disease, hyperlipidemia, cardiovascular disease, autoimmune disease (excluding RA) |
| --- | --- |
| Methods used | Stepwise logistic regression |
| Identification of imbalance in covariates | None, since no matching was performed |
| Final predictors in Cox model | Treatment, propensity score |

Abbreviations: *bDMARD* biologic disease-modifying antirheumatic drug, *COPD* chronic obstructive pulmonary disease, *csDMARD* conventional synthetic disease-modifying antirheumatic drug, *IV* intravenous, *MTX* methotrexate, *NSAID* nonsteroidial anti-inflammatory drug, *RA* rheumatoid arthritis

**Table S2.** MarketScan: propensity score–matched analysis

| Independent predictors considered for propensity score generation | Sex, age at index date, year of index date, any bDMARD, csDMARDs, MTX, IV antibiotics, corticosteroids, NSAIDs, hypertension, diabetes, hospitalized infections, malignancy, lymphoma, COPD, asthma, chronic kidney disease, leukopenia, neutropenia, peripheral artery disease, hyperlipidemia, cardiovascular disease, autoimmune disease (excluding RA) |
| --- | --- |
| Methods used | Stepwise logistic regression |
| Final independent predictors included in propensity score generation | Sex, age at index date, year of index date, any bDMARD, csDMARDs, MTX, IV antibiotics, corticosteroids, NSAIDs, hypertension, diabetes, hospitalized infections, malignancy, lymphoma, asthma, leukopenia, neutropenia, hyperlipidemia, cardiovascular disease, autoimmune disease (excluding RA) |
| Method used for detection of covariate imbalance | Cohen’s D (standardized difference of mean) |
| Variable imbalanced | Any bDMARD |
| Independent predictors considered for propensity score generation | Sex, age at index date, year of index date, csDMARDs, MTX, IV antibiotics, corticosteroids, NSAIDs, hypertension, diabetes, hospitalized infections, malignancy, lymphoma, asthma, leukopenia, neutropenia, hyperlipidemia, cardiovascular disease, autoimmune disease (excluding RA) |
| Method used | Stepwise logistic regression |
| Final independent predictors included in propensity score generation | Sex, age at index date, year of index date, csDMARDs, MTX, IV antibiotics, corticosteroids, NSAIDs, hypertension, diabetes, hospitalized infections, lymphoma, asthma, leukopenia, neutropenia, cardiovascular disease, autoimmune disease (excluding RA) |
| Method used for detection of covariate imbalance | Cohen’s D (standardized difference of mean) |
| Variable imbalanced | None |
| Final model-independent predictors | Treatment, any bDMARD |

Abbreviations: *bDMARD* biologic disease-modifying antirheumatic drug, *COPD* chronic obstructive pulmonary disease, *csDMARD* conventional synthetic disease-modifying antirheumatic drug, *IV* intravenous, *MTX* methotrexate, *NSAID* nonsteroidial anti-inflammatory drug, *RA* rheumatoid arthritis

**Table S3.** PharMetrics: propensity score–adjusted analysis

| Independent predictors considered for propensity score generation | Sex, age at index date, year of index date, any bDMARD, csDMARDs, MTX, IV antibiotics, corticosteroids, NSAIDs, hypertension, diabetes, hospitalized infections, malignancy, lymphoma, COPD, asthma, chronic kidney disease, leukopenia, neutropenia, peripheral artery disease, hyperlipidemia, cardiovascular disease, autoimmune disease (excluding RA) |
| --- | --- |
| Methods used | Stepwise logistic regression |
| Identification of imbalance in covariates | None, since no matching was performed |
| Final predictors in Cox model | Treatment, propensity score |

Abbreviations: *bDMARD* biologic disease-modifying antirheumatic drug, *COPD* chronic obstructive pulmonary disease, *csDMARD* conventional synthetic disease-modifying antirheumatic drug, *IV* intravenous, *MTX* methotrexate, *NSAID* nonsteroidial anti-inflammatory drug, *RA* rheumatoid arthritis

**Table S4.** PharMetrics: propensity score–matched analysis

| Independent predictors considered for propensity score generation | Sex, age at index date, year of index date, any bDMARD, csDMARDs, MTX, IV antibiotics, corticosteroids, NSAIDs, hypertension, diabetes, hospitalized infections, malignancy, lymphoma, COPD, asthma, chronic kidney disease, leukopenia, neutropenia, peripheral artery disease, hyperlipidemia, cardiovascular disease, autoimmune disease (excluding RA) |
| --- | --- |
| Methods used | Stepwise logistic regression |
| Final independent predictors included in propensity score generation | Sex, age at index date, year of index date, any bDMARD, csDMARDs, MTX, IV antibiotics, corticosteroids, NSAIDs, hypertension, diabetes, hospitalized infections, malignancy, lymphoma, COPD, asthma, leukopenia, hyperlipidemia, cardiovascular disease, autoimmune disease (excluding RA) |
| Method used for detection of covariate imbalance | Cohen’s D (standardized difference of mean) |
| Variable imbalanced | Any bDMARD |
| Independent predictors considered for propensity score generation | Sex, age at index date, year of index date, csDMARDs, MTX, IV antibiotics, corticosteroids, NSAIDs, hypertension, diabetes, hospitalized infections, malignancy, lymphoma, COPD, asthma, leukopenia, hyperlipidemia, cardiovascular disease, autoimmune disease (excluding RA) |
| Method used | Stepwise logistic regression |
| Final independent predictors included in propensity score generation | Sex, age at index date, year of index date, csDMARDs, MTX, IV antibiotics, corticosteroids, hypertension, diabetes, hospitalized infections, malignancy, lymphoma, COPD, asthma, leukopenia, cardiovascular disease, autoimmune disease (excluding RA) |
| Method used for detection of covariate imbalance | Cohen’s D (standardized difference of mean) |
| Variable imbalanced | None |
| Final model-independent predictors | Treatment, any bDMARD |

Abbreviations: *bDMARD* biologic disease-modifying antirheumatic drug, *COPD* chronic obstructive pulmonary disease, *csDMARD* conventional synthetic disease-modifying antirheumatic drug, *IV* intravenous, *MTX* methotrexate, *NSAID* nonsteroidial anti-inflammatory drug, *RA* rheumatoid arthritis

**Table S5.** Optum: propensity score–adjusted analysis

| Independent predictors considered for propensity score generation | Sex, age at index date, year of index date, any bDMARD, csDMARDs, MTX, IV antibiotics, corticosteroids, NSAIDs, hypertension, diabetes, hospitalized infections, malignancy, lymphoma, COPD, asthma, chronic kidney disease, leukopenia, neutropenia, peripheral artery disease, hyperlipidemia, cardiovascular disease, autoimmune disease (excluding RA) |
| --- | --- |
| Methods used | Stepwise logistic regression |
| Identification of imbalance in covariates | None, since no matching was performed |
| Final predictors in Cox model | Treatment, propensity score |

Abbreviations: *bDMARD* biologic disease-modifying antirheumatic drug, *COPD* chronic obstructive pulmonary disease, *csDMARD* conventional synthetic disease-modifying antirheumatic drug, *IV* intravenous, *MTX* methotrexate, *NSAID* nonsteroidial anti-inflammatory drug, *RA* rheumatoid arthritis

**Table S6.** Optum: propensity score–matched analysis

| Independent predictors considered for propensity score generation | Sex, age at index date, year of index date, any bDMARD, csDMARDs, MTX, IV antibiotics, corticosteroids, NSAIDs, hypertension, diabetes, hospitalized infections, malignancy, lymphoma, COPD, asthma, chronic kidney disease, leukopenia, neutropenia, peripheral artery disease, hyperlipidemia, cardiovascular disease, auto-immune disease (excluding RA) |
| --- | --- |
| Methods used | Stepwise logistic regression |
| Final independent predictors included in propensity score generation | Sex, age at index date, year of index date, any bDMARD, csDMARDs, MTX, IV antibiotics, corticosteroids, diabetes, hospitalized infections, malignancy, lymphoma, chronic kidney disease, leukopenia, neutropenia, cardiovascular disease, autoimmune disease (excluding RA) |
| Method used for detection of covariate imbalance | Cohen’s D (standardized difference of mean) |
| Variable imbalanced | Any bDMARD |
| Independent predictors considered for propensity score generation | Sex, age at index date, year of index date, csDMARDs, MTX, IV antibiotics, corticosteroids, diabetes, hospitalized infections, malignancy, lymphoma, chronic kidney disease, leukopenia, neutropenia, cardiovascular disease, autoimmune disease (excluding RA) |
| Method used | Stepwise logistic regression |
| Final independent predictors included in propensity score generation | Sex, age at index date, year of index date, csDMARDs, MTX, IV antibiotics, corticosteroids, diabetes, malignancy, lymphoma, leukopenia, neutropenia, cardiovascular disease, autoimmune disease (excluding RA) |
| Method used for detection of covariate imbalance | Cohen’s D (standardized difference of mean) |
| Variable imbalanced | Cardiovascular disease |
| Independent predictors considered for propensity score generation | Sex, age at index date, year of index date, csDMARDs, MTX, IV antibiotics, corticosteroids, diabetes, malignancy, lymphoma, leukopenia, neutropenia, autoimmune disease (excluding RA) |
| Method used | Stepwise logistic regression |
| Final independent predictors included in propensity score generation | Sex, age at index date, year of index date, csDMARDs, MTX, IV antibiotics, corticosteroids, diabetes, malignancy, lymphoma, leukopenia, neutropenia, autoimmune disease (excluding RA) |
| Method used for detection of covariate imbalance | Cohen’s D (standardized difference of mean) |
| Variable imbalanced | None |
| Final model-independent predictors | Treatment, any bDMARD, cardiovascular disease |

Abbreviations: *bDMARD* biologic disease-modifying antirheumatic drug, *COPD* chronic obstructive pulmonary disease, *csDMARD* conventional synthetic disease-modifying antirheumatic drug, *IV* intravenous, *MTX* methotrexate, *NSAID* nonsteroidial anti-inflammatory drug, *RA* rheumatoid arthritis

**Table S7.** Demographics and baseline characteristics of patients in the unmatched abatacept and other b/tsDMARDs groups

|  | MarketScan | | PharMetrics | | Optum | |
| --- | --- | --- | --- | --- | --- | --- |
|  | Abatacept  (n=19,170) | Other b/tsDMARDs*  (n=55,261) | Abatacept  (n=13,590) | Other b/tsDMARDs*  (n=40,751) | Abatacept  (n=4201) | Other b/tsDMARDs*  (n=13,846) |
| Female, % | 82 | 74 | 81 | 72 | 83 | 72 |
| Age in years at index date, mean (SD) | 55 (13) | 53 (13) | 54 (12) | 51 (13) | 52 (11) | 49 (12) |
| Co-morbid conditions during the baseline period, % | | | | | | |
| Malignancy | 5.2 | 6.1 | 4.9 | 5.4 | 4.6 | 4.6 |
| Cardiovascular disease^†^ | 24 | 20 | 23 | 20 | 22 | 18 |
| Hospitalized infections | 3.6 | 3.0 | 4.0 | 3.4 | 4.2 | 3.4 |
| Other autoimmune diseases^‡^ | 17 | 21 | 19 | 23 | 20 | 25 |
| Co-medications^§^ , % | | | | | | |
| csDMARDs | 59 | 41 | 66 | 39 | 71 | 47 |
| b/tsDMARDs | 49 | 14 | 53 | 14 | 58 | 19 |
| Glucocorticoids | 57 | 45 | 64 | 46 | 73 | 56 |

*Excludes abatacept. ^†^ includes: ischemic heart disease; diseases of pulmonary circulation; other forms of heart disease; cerebrovascular disease; diseases of arteries, arterioles, and capillaries; diseases of veins and lymphatics; other diseases of the circulatory system. ^‡^includes: psoriatic arthropathy; other psoriasis; diabetes mellitus; multiple sclerosis; systemic lupus erythematosus; vitiligo; toxic diffuse goiter without mention of thryrotoxic crisis or storm; chronic lymphocytic thyroiditis; corticoadrenal insufficiency; acquired hemolytic anemias; immune thrombocytopenic purpura; chronic glomerulonephritis; cirrhosis of liver without mention of alcohol; celiac disease; regional enteritis; ulcerative enterocolitis; postinflammatory pulmonary fibrosis; giant cell arteritis; sicca syndrome; systemic sclerosis; alopecia areata; urticaria. ^§^includes medications taken within 180 days before the index date.

Abbreviations: *b/tsDMARDs* biologic or targeted synthetic disease-modifying antirheumatic drugs, *csDMARDs* conventional synthetic disease-modifying antirheumatic drugs, *SD* standard deviation

**Table S8.** Index treatment in the unmatched other b/tsDMARDs group*

| Treatment, % | MarketScan  (n=55,261) | PharMetrics  (n=40,751) | Optum  (n=13,846) |
| --- | --- | --- | --- |
| Etanercept | 32 | 39 | 40 |
| Adalimumab | 26 | 30 | 29 |
| Infliximab | 20 | 15 | 13 |
| Rituximab | 12 | 9 | 7 |
| Certolizumab pegol | 3 | 2 | 4 |
| Golimumab | 3 | 2 | 4 |
| Tocilizumab | 3 | 2 | 2 |
| Tofacitinib | 1 | 1 | 1 |
| Anakinra | <1 | 1 | 1 |

*Excludes abatacept.

Abbreviation: *b/tsDMARD* biologic or targeted synthetic disease-modifying antirheumatic drug

**Table S9.** IRs per 1000 py (95% CI) for total malignancy (one ICD-9-CM code)

|  | MarketScan | | PharMetrics | | Optum | |
| --- | --- | --- | --- | --- | --- | --- |
|  | Abatacept  (n=19,170) | Other b/tsDMARDs*  (n=55,261) | Abatacept  (n=13,590) | Other b/tsDMARDs*  (n=40,751) | Abatacept  (n=4201) | Other b/tsDMARDs*  (n=13,846) |
| Follow-up^†^, years (mean) | 2.0 | 2.2 | 2.4 | 2.7 | 2.3 | 2.4 |
| Age, years | IR (95% CI) | | | | | |
| Overall | 44 (42–47) | 36 (35–38) | 40 (37–43) | 32 (31–33) | 37 (32–42) | 29 (27–31) |
| 18–64 | 35 (32–37) | 29 (28–30) | 33 (31–36) | 27 (26–29) | 33 (29–38) | 27 (24–29) |
| 65–74 | 83 (73–95) | 77 (71–84) | 86 (72–100) | 78 (70–86) | 68 (43–103) | 53 (41–67) |
| ≥75 | 100 (85–116) | 93 (83–103) | 90 (70–114) | 96 (82–113) | 104 (57–174) | 67 (44–98) |

*Excludes abatacept. ^†^Includes a latency period of 180 days.

Abbreviations: *b/tsDMARDs* biologic or targeted synthetic disease-modifying antirheumatic drugs, *CI* confidence interval, *ICD-9-CM* International Classification of Diseases, Ninth Revision, Clinical Modification*, IR* incidence rate, *py* person-years

**Table S10.** Overall number of events/number of py and IRs/1000 py (95% CI) of malignancies (one ICD-9-CM code)

|  |  | MarketScan | | | PharMetrics | | | | Optum | | |
| --- | --- | --- | --- | --- | --- | --- | --- | --- | --- | --- | --- |
|  |  | Abatacept | Other b/tsDMARDs* | | Abatacept | | Other b/tsDMARDs* | | Abatacept | | Other b/tsDMARDs* |
| Lung cancer | Events/py | 107/29,577 | 287/93,318 | 74/23,183 | | | 199/81,247 | | 11/6708 | | 43/24,718 |
|  | IR/1000 py (95% CI) | 4 (3–4) | 3 (3–3) | 3 (3–4) | | | 2 (2–3) | | 2 (1–3) | | 2 (1–2) |
| Lymphoma | Events/py | 74/29,473 | 258/91,604 | 73/23,062 | | | 208/80,082 | | 15/6680 | | 47/24,360 |
|  | IR/1000 py (95% CI) | 3 (2–3) | 3 (2–3) | 3 (2–4) | | | 3 (2–3) | | 2 (1–4) | | 2 (1–3) |
| Breast cancer | Events/py | 178/28,862 | 424/91,861 | 132/22,677 | | 356/79,938 | | 38/6536 | | 102/24,337 | |
|  | IR/1000 py (95% CI) | 6 (5–7) | 5 (4–5) | 6 (5–7) | | 4 (4–5) | | 6 (4–8) | | 4 (3–5) | |
| Non-melanoma skin cancer | Events/py | 620/27,756 | 1527/88,569 | 413/22,043 | | 1176/77,738 | | 123/6384 | | 334/23,588 | |
|  | IR/1000 py (95% CI) | 22 (21–24) | 17 (16–18) | 19 (17–21) | | 15 (14–16) | | 19 (16–23) | | 14 (13–16) | |
| Total malignancy | Events/py | 1132/25,486 | 2933/80,929 | 804/20,149 | | 2293/71,360 | | 218/5914 | | 630/21,879 | |
|  | IR/1000 py (95% CI) | 44 (42–47) | 36 (35–38) | 40 (37–43) | | 32 (31–33) | | 37 (32–42) | | 29(27–31) | |

*Excludes abatacept.

Abbreviations: *b/tsDMARDs* biologic or targeted synthetic disease-modifying antirheumatic drugs, *CI* confidence interval, *ICD-9-CM*  International Classification of Diseases, Ninth Revision, Clinical Modification*, IR* incidence rate, *py* person-years

**Table S11.** Overall number of events/number of py and IRs/1000 py (95% CI) of infections (one ICD-9-CM code)

|  |  | MarketScan | | PharMetrics | | Optum | |
| --- | --- | --- | --- | --- | --- | --- | --- |
|  |  | Abatacept | Other b/tsDMARDs* | Abatacept | Other b/tsDMARDs* | Abatacept | Other b/tsDMARDs* |
| Hospitalized infections^†^ | Events/py | 463/15,715 | 1532/48,227 | 340/12,030 | 1185/41,746 | 102/3692 | 340/12,313 |
|  | IR/1000 py (95% CI) | 29 (27–32) | 32 (30–33) | 28 (25–31) | 28 (27–30) | 28 (22–34) | 28 (25–31) |
| Opportunistic infections | Events/py | 1229/14,427 | 3244/44,292 | 958/10,984 | 2800/38,085 | 323/3315 | 919/11,224 |
|  | IR/1000 py (95% CI) | 85 (80–90) | 73 (71–76) | 87 (82–93) | 74 (71–76) | 97 (87–109) | 82 (77–87) |
| Tuberculosis | Events/py | 38/16,552 | 122/50,240 | 29/12,685 | 75/43,561 | 18/3858 | 22/12,816 |
|  | IR/1000 py (95% CI) | 2 (2–3) | 2 (2–3) | 2 (2–3) | 2 (1–2) | 5 (3–7) | 2 (1–3) |

*Excludes abatacept. ^†^ Primary diagnosis.

Abbreviations: *b/tsDMARDs* biologic or targeted synthetic disease-modifying antirheumatic drugs, *CI* confidence interval, *ICD-9-CM*  International Classification of Diseases, Ninth Revision, Clinical Modification*, IR* incidence rate, *py* person-years

**Table S12.** IRs per 1000 py (95% CI) for hospitalized infections (primary diagnosis; one ICD-9-CM code)

|  | MarketScan | | PharMetrics | | Optum | |
| --- | --- | --- | --- | --- | --- | --- |
|  | Abatacept  (n=19,170) | Other b/tsDMARDs*  (n=55,261) | Abatacept  (n=13,590) | Other b/tsDMARDs*  (n=40,751) | Abatacept  (n=4201) | Other b/tsDMARDs*  (n=13,846) |
| Follow-up, years (mean) | 2.0 | 2.2 | 2.4 | 2.7 | 2.3 | 2.4 |
| Age, years | IR (95% CI) | | | | | |
| Overall | 29 (27–32) | 32 (30–33) | 28 (25–31) | 28 (27–30) | 28 (23–34) | 28 (25–31) |
| 18–64 | 23 (21–26) | 26 (24–28) | 24 (21–27) | 25 (23–26) | 27 (22–33) | 25 (22–28) |
| 65–74 | 48 (39–59) | 55 (49–62) | 49 (38–63) | 51 (44–60) | 29 (11–63) | 42 (29–60) |
| ≥75 | 71 (55–88) | 83 (72–96) | 61 (41–87) | 90 (73–111) | 43 (9–126) | 106 (65–164) |

*Excludes abatacept.

Abbreviations: *b/tsDMARDs* biologic or targeted synthetic disease-modifying antirheumatic drugs, *CI* confidence interval, *ICD-9-CM*  International Classification of Diseases, Ninth Revision, Clinical Modification*, IR* incidence rate, *py* person-years
